# The motor vertical in the absence of gravicentric cues

**DOI:** 10.1038/s41526-020-0098-8

**Published:** 2020-03-03

**Authors:** Otmar Bock, Nils Bury

**Affiliations:** 1 Institute of Exercise Training and Sport Informatics, German Sport University, 50927 Köln, Germany; 2 Department of Psychology, York University, Toronto, ON Canada

**Keywords:** Neuroscience, Neuroscience, Human behaviour, Human behaviour

## Abstract

When participants are asked to flip an omnidirectional switch “down”, the direction of their responses depends mainly on gravicentric, less so on egocentric and least on visual cues about the vertical (Lackner and DiZio, Exp. Brain Res. 130:2−26, 2000). Here we evaluate response direction when gravicentric cues are not available. Participants flipped an omnidirectional switch “down” when gravito-inertial force acted orthogonally to the response plane on earth (session E), and when it was near zero during parabolic flights (session P). We found that the relative weight of visual cues was similar in both sessions, and it was similar to that in an earlier study where participants stood upright. Across all three data sets, the weight of visual cues averaged 0.09. The relative weight of egocentric cues was also similar in both sessions, averaging 0.87; however, it was significantly lower in the earlier study with upright participants, where it averaged 0.43. We further found that informative and noninformative tactile stimulation had no substantial effects on response direction, which suggests that the earlier reported anchoring effect of tactile signals for the perceived vertical may not extend to the motor vertical. We conclude that the absence of gravicentric cues is compensated by a higher weight of egocentric cues, but not by a higher weight of visual cues. As a consequence, astronauts, divers and persons who work on ground in a horizontal body posture may mishandle equipment because of their strong reliance on egocentric cues.

## Introduction

Our perception of the vertical can utilize three types of information, *visual* cues about the orientation of familiar objects such as walls and trees,^1^ *gravicentric* cues about the direction of the gravito-inertial force (GIF)^2^ and *egocentric* cues about the orientation of our own body.^3^ A number of studies investigated the respective roles of these three cues by tilting participants and/or their visual surrounds with respect to the GIF, and then asking them to indicate their perceived direction of gravity,^4,5^ their perceived body orientation,^6,7^ or first the one and then the other.^8–10^ All in all, participants’ responses were reasonably accurate, which documents that humans are able to access gravicentric and egocentric information when they are asked to do so. This research left open, however, which information we use for our perception when no experimenter tells us to use only one specific source of information. In other words, this research lacks ecological validity.

A smaller number of studies did not instruct participants to use any specific cues about the vertical, and rather asked them to “indicate the apparent vertical”^11–14^ to “straighten” an object or their own body,^15^ to tell whether an object “appeared normal”,^9^ to pronounce letters whose identity depends on the vertical^16^ or to indicate the shape of objects whose shape depends on the vertical.^17^ By avoiding cue-specific instructions, these studies came closer to situations of real life; their ecological validity is therefore higher than in the studies mentioned before. It was found that in the absence of cue-specific instructions, the perceived vertical was *not* aligned with one of the available cue types, i.e., it did not follow the “winner takes all” principle. Rather, the perceived vertical was a *weighted sum* of visual, gravicentric and egocentric cues.^16–18^

We reasoned that an internal representation of the vertical is needed not only for perception but also for motor control, e.g., when we lift a cup of coffee “up”, flip a switch “down”, or maintain an “upright” body posture. We further reasoned that the perceptual and the motor system might access distinct internal representations of the vertical: perception and action can obey different rules^19,20^ (see, however, refs. ^21,22^), follow different neural pathways,^23,24^ and therefore might also use different representations of the vertical.^25^ We coined the term “motor vertical” for the internal representation that is used by the arm motor system.^26^ For reasons of ecological validity, we operationalized the motor vertical as the direction of arm movement when a person intends to move the arm “down” in the absence of cue-specific instructions. We were not interested in the direction of arm movement when a person is instructed to move the arm parallel to the GIF, parallel to the own body axis, or parallel to a visual reference.

Past literature provides little knowledge about the motor vertical. Two studies addressed this topic indirectly, by evaluating the up−down asymmetry of hand velocity profiles. They observed that under conditions of body tilt^27^ or visual tilt,^28^ this asymmetry was not aligned with the true gravicentric, egocentric or visual vertical but rather assumed an intermediate direction. This outcome is compatible with the view that the motor vertical, just like the perceived vertical, is a weighted sum of all three cue types.

In our own research, we asked participants to flip a switch “down”, once while they stood upright and once while they were tilted with respect to the GIF. We designed and positioned the switch such that it could be flipped into any direction in the participants’ frontal plane, and such that it conveyed a palpable click whenever it was flipped.

When instructing our participants, we did not explain what we meant by “down”. We did not ask them to use any particular cues about “down”, and we did not even mention the existence of such cues. If participants voiced queries, we told them to respond in accordance with “whatever you feel to be ‘down’”. Participants were therefore free to specify “down” in any way they felt to be adequate.

In our initial experiments,^26^ *visual* cues about the vertical were absent; we found that participants flipped the switch in a direction that corresponded to the weighted sum of gravicentric and egocentric cues. The relative weight of gravicentric cues was 0.16 and that of egocentric cues was 1–0.16 = 0.84 when the responding arm was supported against the GIF by slings.^26^ The relative weight of gravicentric cues was substantially higher (0.77) and that of egocentric cues was accordingly lower (0.23) when the arm was unsupported (Bury and Bock, in preparation), from which we concluded that the weight of gravicentric cues is higher if the GIF act on the responding arm. For reasons of ecological validity, we decided to keep the responding arm unsupported in our further research.

Our next experiment introduced visual cues that were congruent with the direction of the GIF. Participants’ responses indicated that the combined weight of visual and gravicentric cues was 0.62, and that the weight of egocentric cues accordingly was 0.38.^29^ A subsequent study varied visual and gravicentric cues independently. We found that the weight of visual cues alone was 0.13, that of gravicentric cues alone was 0.52, and that of egocentric cues therefore was 0.35.^30^ Note that the sum of visual and gravicentric cues in the latter experiment fits well with the combined visual-gravicentric weight in the former experiment. Note further that the weight of visual cues alone was surprisingly low, given the many earlier accounts about visual dominance (e.g., ref. ^31^). Our finding therefore confirms that vision does not dominate in all experimental paradigms,^32,33^ including a paradigm exploring the perceived vertical.^17^

The present study continues our line of research by evaluating the motor vertical when gravicentric cues are not available. To this end, we tested participants in a kneeling posture, with their face directed towards the floor. Each person was tested once on earth (condition E) and once during the short-term weightlessness of parabolic flight (condition P). As in our earlier work, participants operated a switch that could be deflected in any direction of their frontal plane. Unlike in our earlier work, however, the GIF did not serve as a cue since it acted orthogonally to the response plane (E) or was nearly absent (P). We reasoned that participants might compensate the absence of gravicentric cues by giving a higher weight to visual cues. Alternatively, they might compensate by giving a higher weight to egocentric cues. As a third alternative, they might give a higher weight both to visual and to egocentric cues.

Note that conditions E and P are not equivalent for several reasons. First, ground reaction forces are present in E but not in P. Those forces are likely to stabilize the participants’ body with respect to the experimental apparatus, and they may serve as a cognitive anchor^34^ which refines participants’ internal representation of space. Second, muscular tone is likely to be lower in P compared to E,^35^ which is known to degrade the limb position sense.^36,37^ We reasoned that these differences regarding body stability, cognitive anchoring and limb position sense might be reflected by a higher response variability in P compared to E. We did not expect that these differences will affect mean response directions, i.e., that they will change the cue weights.

Based on the above considerations, we formulated the following research questions:

Q1: Will the absence of gravicentric cues be compensated by a higher weight of visual cues, of egocentric cues, or both?

Q2: Will this compensation be similar when noninformative GIF is present versus absent?

Q3: Will the absence of gravicentric cues result in a higher intraindividual response variability?

Q4: Will that variability be similar when noninformative GIF is present versus absent?

Our earlier work included conditions in which participants grasped a handhold with the nonresponding hand, thus adding tactile cues about the vertical. We found no^29^ or only marginal effects^30^ of those cues, but we nevertheless decided to re-evaluate their role in the present study. This decision was based on the earlier finding that in microgravity, even noninformative tactile signals can improve perception of the own body axis.^38^ Tactile signals were produced by pressure on the chest in that study, and possibly served as a cognitive anchor.^34^ This led us to the following question:

Q5: Will the weight of egocentric cues depend on the presence of tactile cues and on their type (noninformative, informative and confirmatory, informative and conflicting)?

## Results

**Fig. 1:**
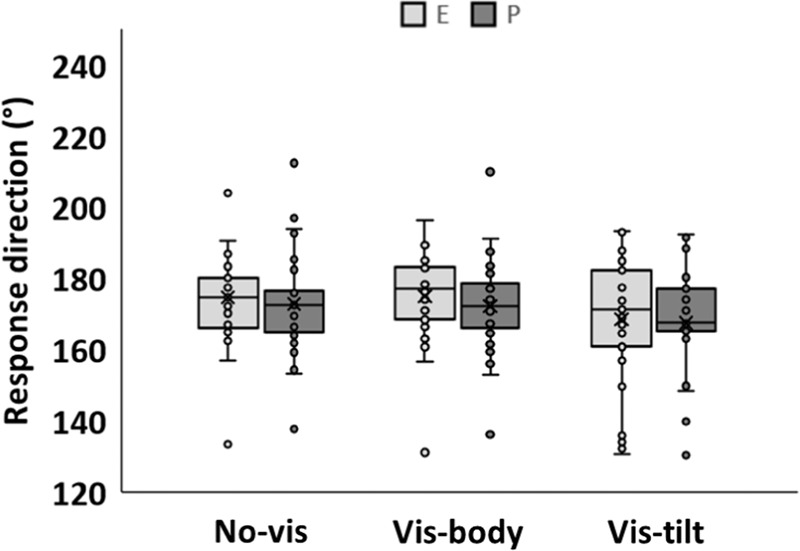
Response direction as a function of visual condition. Data were registered once in parabolic flight (session P), and once while participants were lying with the torso prone on ground (session E). In *vis-body*, the display was aligned with the participants’ long body axis while in *vis-tilt*, it was rotated 45° clockwise with respect to that axis. Each dot is the mean across trials of one participant. Boxes represent ±1 interquartile range, whiskers ±1.5 interquartile range, lines are medians and “x” are means. ANOVA of Session × Condition yielded significance only for the factor Condition.

Figure 1 shows our participants’ response directions in sessions E and P, separately for three visual conditions. In condition *no-vis*, no visual cues about “down” were provided; in condition *vis-body*, visual cues were oriented parallel to the participants’ long body axis; in condition *vis-tilt*, visual cues were rotated 45° clockwise with respect to that axis. For more details about the visual conditions, see the Methods section and Fig. 4. According to Fig. 1, response directions deviated somewhat from the long body axis (i.e., 180°) in *vis-body*, deviated slightly more in *no-vis*, and even more so in *vis-tilt*. This pattern was similar for E and P. Accordingly, ANOVA of Session × Condition yielded significance only for the factor Condition, as shown in Table 1. Post-hoc decomposition (Fisher’s LSD) revealed significant differences only between *vis-tilt* and the other two conditions (both *p* < 0.025).

**Table 1:** Outcome of Session × Condition ANOVA for response direction (this analysis is based on the data from all participants).

	df	F	*p*
Session	1,28	1.21	0.279
Condition	2,56	4.76	0.012
S × C	2,56	0.17	0.837

**Fig. 2:**
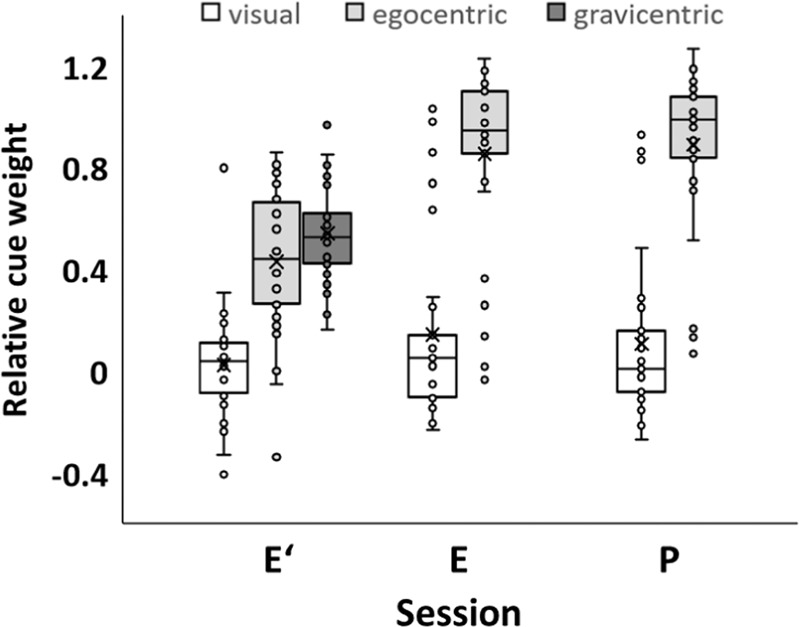
Relative weight of visual, egocentric and gravicentric cues. Data were registered when participants were upright on ground (session E′; data from an earlier study^31^), lying with the torso prone on ground (session E) or free-floating in parabolic flight (session P). Symbols as in Fig. 1. T tests yielded no significant differences between visual cue weights in E′ and E, in E′ and P, or in E and P, as well as no significant differences between egocentric cue weights in E and P. Significance emerged only for the differences between egocentric cue weights in E′ and E as well as E′ and P.

The relative weight of visual cues *w*_v_ was calculated from response directions as described in Methods. The white boxes in Fig. 2 illustrate that across individuals, *w*_v_ was quite low in our earlier study^30^ where participants stood upright on ground (session E′), and was also low in present session E and P. *T* tests yielded no significant differences between E′ and E (*t*(53) = 1.45; *p* > 0.05), between E′ and P (*t*(53) = 1.11; *p* > 0.05) or between E and P (*t*(28) = 0.55; *p* > 0.05). Across all three sessions, *w*_v_ averaged 0.09.

The relative weight of egocentric cues *w*_e_ was calculated from response directions as also described in Methods. The light gray boxes in Fig. 2 show that across individuals, *w*_e_ was substantially lower in E′ compared to P and E. *T* tests yielded significant differences between E′ and E (*t*(53) = 4.74; *p* < 0.001) between E′ and P (*t*(53) = 5.57; *p* < 0.001) but not between E and P (*t*(28) = 0.55; *p* > 0.05). *w*_e_ averaged 0.43 in E′, and 0.87 in P and E. Further from Fig. 2, a few participants exhibited the opposite trend, with high *w*_v_ and low *w*_e_. This was the case for five persons in E and three persons in P.

The role of tactile cues was investigated by testing participants while they grasped a handhold that was oriented parallel to their long body axis (condition *grip-body*), while they grasped a handhold that was rotated 45° clockwise with respect to that axis (condition *grip-tilt*), and while a kidney belt provided noninformative stimulation (condition *belt*); for details, see Methods. ANOVA of Session × Condition × Handhold yielded no significance, as shown in Table 2. Similarly, ANOVA of Session × Condition × Belt also revealed no significance, as shown in Table 3.

**Table 2:** Outcome of Session × Condition × Handhold ANOVA for normalized response direction with and without grasping a handhold (this analysis is based on the data from all participants).

	df	F	*p*
Session	1,27	3.05	0.091
Condition	1,27	1.47	0.234
S × C	1,27	0.03	0.857
Handhold	1,27	2.69	0.112
S × H	1,27	0.12	0.729
C × H	1,27	0.04	0.837
S × C × H	1,27	0.93	0.341

**Table 3:** Outcome of Session × Condition × Belt ANOVA for response direction (this analysis is based on the data from those 14 participants who were tested with a belt).

	df	F	*p*
Session	1,13	0.03	0.848
Condition	1,13	1.96	0.184
S × C	1,13	0.46	0.508
Belt	1,13	0.07	0.790
S × B	1,13	0.02	0.890
C × B	1,13	1.44	0.250
S × C × B	1,13	0.50	0.490

**Fig. 3:**
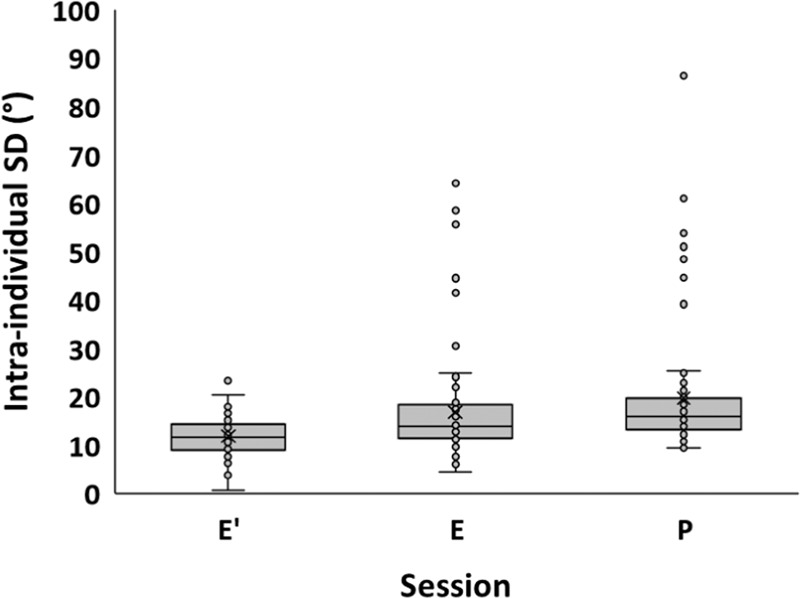
Intraindividual standard deviations. Data are from condition E′ (earlier study with different participants^31^), E and P. Symbols as in Fig. 1. ANOVAs of Session × Condition yielded significance only for the factor Session.

The standard deviation of response directions was compared by ANOVAs of Session × Condition, separately for each pair of sessions (E and P, E and E′, P and E′). Significance emerged in all three ANOVAs only for the factor Session, as shown in Tables 4–6. Figure 3 illustrates that response variability was lowest in E′, higher in E and higher still in P.

**Table 4:** Outcome of Session × Condition ANOVA for the variability of response direction (Sessions E and P are compared).

	df	F	*p*
Session	1,28	6.20	0.019
Condition	2,56	2.10	0.131
S × C	2,56	0.54	0.583

**Table 5:** Outcome of Session × Condition ANOVA for the variability of response direction (Sessions E and E′ are compared).

	df	F	p
Session	1,54	6.93	0.011
Condition	2,108	1.82	0.166
S × C	2,108	0.23	0.789

**Table 6:** Outcome of Session × Condition ANOVA for the variability of response direction (Sessions P and E′ are compared).

	df	F	p
Session	1,54	12.06	0.001
Condition	2,108	2.13	0.124
S * C	2,108	0.89	0.412

## Discussion

Continuing a series of experiments, the present study evaluated the role of the GIF for the motor vertical. Participants were asked to flip a switch “down” once while the GIF acted orthogonally to the response plane (session E), and once while the GIF was almost nil (session P). We addressed the following questions.

*Q1: Will the absence of gravicentric cues be compensated by a higher weight of visual cues, of egocentric cues, or both?*

Comparing the present data with those in an earlier study where participants stood upright,^30^ we found that in the absence of gravicentric cues, the relative weight of egocentric cues was dramatically higher while the relative weight of visual cues changed little. To explain why then latter did not change, we recourse to the maximum-likelihood hypothesis of multisensory integration.^32^ According to that hypothesis, different sensory signals about a given external phenomenon are combined towards a single percept by weighing each signal with respect to its informative value. If so, our finding might indicate that the reliability of visual cues about the vertical is lower in the absence of the GIF, possibly because visuo-spatial processing in the vestibular nuclei,^39^ the vestibulocerebellum^40^ and the vestibular cortex^41^ is impaired when matching vestibular signals are not available.

*Q2: Will this compensation be similar when noninformative GIF is present versus absent?*

Comparing the two sessions of the present study, we found that the weight of visual cues was similar in E and P. In consequence, the weight of egocentric cues (*w*_e_ = 1 – *w*_v_) was similar in E and P as well. Noninformative GIF therefore seems not to have a systematic influence on the motor vertical.

*Q3: Will the absence of gravicentric cues result in a higher intraindividual response variability, and*

*Q4: will that variability be similar when noninformative GIF is present versus absent?*

It is useful to consider these two questions together. We reasoned (see Introduction) that response variability will not be the same across sessions because the GIF may (1) serve as a cognitive anchor for the motor vertical, (2) prevent low muscular tone and therefore poor limb position sense, or (3) stabilize the body with respect to the experimental apparatus. All three views could explain why response variability was lower in E than in P. However, the three views make different predictions regarding response variability in E compared to E′. Cognitive anchoring should be similar in E and E′; view (1) therefore predicts similar variability in E and E′. Muscular tone should also be similar in E and E′, or it should be *higher* in E because of the unusual, prone body posture; view (2) therefore predicts similar or lower variability in E compared to E′. Body stability should again be similar in E and E′, or it should be *lower* in E because of the prone body posture; view (3) therefore predicts similar or higher variability in E compared to E′. Only view (3) is compatible with the actual data in Fig. 3, and Table 5, from which we conclude that the observed differences of response variability are more likely related to body stability than to participants’ internal representation of the motor vertical. Note that this conclusion is not challenged by the fact that response variability is affected by the intake of anti-motion sickness drugs:^42,43^ our participants did not take those drugs in either of the two compared conditions, E and E′.

*Q5: Will the weight of egocentric cues depend on the presence of tactile cues and on their type (noninformative, informative and confirmatory, informative and conflicting)?*

Orientation of a handgrip and presence of a kidney belt had no substantial effects on response direction in E or in P. We therefore found no support for an influence of informative or noninformative tactile stimuli on the motor vertical. This seems at odds with an earlier study where an influence of noninformative tactile stimuli was observed during parabolic flight.^38^ However, that earlier study dealt with the perceived rather than with the motor vertical, and the discrepancy between studies may therefore once again reflect the known differences between perception and action.^19,20^ In any case, the observed ineffectiveness of tactile cues may be explained with the framework of the maximum-likelihood hypothesis, according to which signals with poor informative value are weighed low during multisensory integration (see above).

Summing up, the present work documents that when gravicentric cues about the motor vertical are absent on earth or during parabolic flight, the weight of egocentric cues is substantially higher while the weight of visual cues remains low. Presence of noninformative GIF, of noninformative tactile stimulation or of informative tactile stimulation had no influence on those weights. Likewise, presence of noninformative GIF had no substantial effect on cue weights. However, noninformative GIF was associated with lower response variability, possibly by way of stabilizing the participants’ body with respect to the experimental apparatus.

The present findings are of practical relevance for astronauts, divers and persons who work on ground in a horizontal body posture. Our data suggest that those persons will rely mainly on an egocentric representation of “down” and therefore may mishandle equipment that is not aligned with their body.

## Methods

### Participants and setup

Thirty right-handed participants (12 female, 18 male; 29 ± 3.7 yrs.) took part in this study, which was preapproved by the Ethics Commission of the German Sport University and conformed to the Declaration of Helsinki. All participants signed a written informed consent statement before testing began, and passed the flight medical examination. They had no prior experience in research on spatial orientation or parabolic flights (except three participants, who had flown once before testing). They had no vestibular, tactile or somatosensory dysfunctions by self-report, and no such dysfunctions were observed by the experimenters during testing. Any refraction errors were corrected by eyeglasses. All participants took the standard medication of scopolamine (0.5–0.7 mg) before the parabolic flight to reduce the susceptibility of motion sickness.

**Fig. 4:**
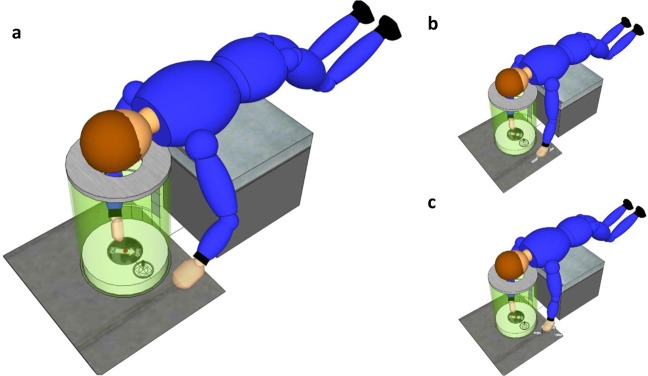
Schematic presentation of the experimental setup. The participants‘ view was constrained by an opaque cylindrical tube (shown here in translucent green in order to illustrate the inside) and by black shrouds (omitted here for clarity). The right (dominant) hand reached into the cylinder to depress the luminated starting button (red). When the starting button was extinguished and the LED ring around the switch lit up (circle near the cylinder wall), participants moved their hand towards the switch, flipped it “down”, and then returned their arm to the starting button. Before the next trial, switch position was changed by rotating the switch about the starting button, i.e., along the tube wall. **a** shows condition *vis-tilt:* tube insight was dimly lit, and participants saw a display rotated 45° with respect to their body axis. In condition *vis-body*, the display was aligned with their body and in condition *no vis* it was not visible. **b** shows condition *vis-tilt* with *grip-body*, and **c** shows condition *vis-tilt* with *grip-tilt*. Condition *vis-tilt* with *belt* is not shown. In session E (on earth), participants’ torso was supported by a box. In session P (parabolic flight), their torso was free-floating and only secured by a loose strap around the waist. At the end of each 22-s microgravity episode, an experimenter pushed participants’ bodies back towards the box for safety reasons.

The hardware was identical to that in our preceding studies.^28,29^ However, participants were tested in different environmental conditions and body postures: first on earth while lying with the torso prone (session E), and 1 day later during the microgravity episodes of parabolic flight (session P). As shown in Fig. 4, participants’ torso was supported by a padded box (session E) or free-floated above that box (session P). Vision was limited by a shroud to a dimly illuminated cylindrical tube of 37 cm diameter and 40 cm length. Located at the base of that tube was a central starting button of 1 cm diameter. At a distance of 11.5 cm from that button was a switch, surrounded by an LED ring of 7.5 cm diameter. The handle of the switch had a diameter of 1 cm diameter and protruded 4 cm into the cylinder. Unlike a conventional toggle switch, which can only be flipped into an “on” or an “off” direction, the switch in our study was omnidirectional: it could be flipped into any direction within the full 360° range in participants‘ frontal plane. It produced a palpable “click” when it was flipped, and returned to the center position when it was released.

Participants were asked to flip it into the direction which they felt to be “down”, without telling them exactly what is “down”. Specifically, we did not tell them that visual, egocentric but not gravicentric cues about “down” will be available. If participants asked us what we meant by “down”, we told them to respond in accordance with “whatever you feel to be ‘down’”. To ensure that participants obey instructions and aim “down” on repeated trials, rather than replicating the motor program they established on the first trial, we changed switch position after each trial. A microprocessor-controlled stepping motor rotated the switch-and-LED assembly about the starting button (i.e., along the tube wall and in the participants’ frontal plane, cf. Fig. 4). Thus, switch-button *distance* remained constant at 11.5 cm but switch-button *direction* varied between −60° and +160°. (Switch directions are expressed in the participants’ body coordinates, with 0° denoting “towards the participants’ head” and 90° denoting “towards the participants’ left side”.) More extreme positions were not included because of biomechanical constraints. Note that switch position is not predicted to provide information about the vertical.

In a baseline condition, no visual cues about “down” were provided (condition *no-vis*). In two other conditions, a schematic drawing of 100 mm diameter was affixed to the inner bottom of the cylinder to provide visual cues about “down”. It consisted of a luminescent arrow with the word “off” written below its tip, and the word “on” written above its base. The arrow was oriented parallel to the participants’ long body axis (condition *vis-body*), or was rotated 45° clockwise with respect to that axis (condition *vis-tilt*). Figure 4 illustrates the setup in condition *vis-tilt*.

The above three conditions were supplemented by three subconditions in which noninformative or informative tactile stimuli were added. The experimenter either attached a kidney belt around the participants’ waist (subcondition *belt*), or asked participants to grasp with their left hand a handhold. The handhold was affixed to the experimental apparatus in a convenient grasping location, had a cylindrical shape, and was oriented parallel to the participants’ response plane. It was either aligned with their long body axis (subcondition *grip-body*), or was rotated 45° clockwise with respect to that axis (subcondition *grip-tilt*).

### General procedures

At the onset of each trial, participants depressed the dimly flashing central starting button with their index finger, which triggered a rotation of the switch-and-LED assembly. The LED ring then lit up in blue for 500 ms as a get-ready signal, and then turned yellow. Participants were instructed to reach for the switch after the color change, to flip the handle “down” with their index finger, and then to return their hand to the starting button. When the switch was flipped within 1500 ms, the LED light extinguished. Otherwise LEDs turned red for 500 ms, reminding participants to act faster on the next trial.

Each experimental condition consisted of 12 trials, each with a different switch position (−60°, −40°, −20°, 0°, 20°, 40°, 60°, 90°, 100°, 120°, 140° and 160°). These positions were presented in the same, quasi-random order to all participants. Since each 22 s microgravity episode of parabolic flight could accommodate six trials, two parabolas were needed to complete all 12 trials.

All participants were tested in the three basic conditions *no-vis*, *vis-body* and *vis-tilt*. Fourteen participants were tested additionally in conditions *no-vis* with *grip-body, no-vis* with *belt, vis-tilt* with *grip-body* and *vis-tilt* with *belt*. The remaining participants were instead tested in conditions *no-vis* with *grip-tilt* and *vis-tilt* with *grip-tilt*. This between-participant design was necessary because grip orientation could not be changed during parabolic flights. One participant from the latter group was not analyzed since he experienced motion sickness during parabolic flight, i.e., there were 15 participants in the latter group. The order of conditions was balanced across participants, separately for the former and for the latter group. Because of scheduling constraints on parabolic-flight campaigns, sessions were administered to all participants in the same order: E on one day, and in P on the next day.

### Data analysis

**Fig. 5:**
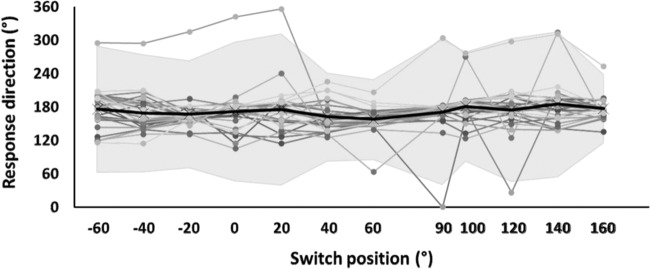
Exemplary data. Direction of all responses registered during parabolic flight in condition *vis-tilt* without a handhold. Each symbol represents one individual response, and each thin line connects the responses of one participant. Bold line and shaded area represent the mean ± 3 standard deviations range which we used to define outliers.

Figure 5 shows original data from an exemplary condition, *vis-tilt*. Switch positions and response directions are expressed in the same, body-centered coordinates (see above). Most responses range between about 110° and 200°, independent of switch position, but there also are some responses well outside that range. We identified such outliers by the “mean ± 3 SD” criterion, where SD is the standard deviation of data from all participants in the respective session, condition and switch position (see shaded area in Fig. 5). Outliers represented 2.3% of responses from session E, and 2.4% from session P, and were excluded from further analyses. Participants in our earlier study^30^ sometimes produced responses that were related to switch position with a slope of about +1 (type-2 responses). We found little evidence for such responses in the present study, and therefore did not analyze them.

We calculated the mean response direction separately for each participant, session and condition. If at least five responses were available, we also calculated their standard deviation. 5.2% of means could not be calculated because participants skipped a condition or because of technical problems. We replaced those missing means by the grand mean across all other participants in the same session and condition, and compensated for the resultant central bias by setting the significance threshold to *p* < 0.025 rather than the usual *p* < 0.05.

Responses while grasping a handhold were available for some participants in *grip-body* and for others in *grip-tilt*. To reduce the effects of interindividual variability when comparing the two handhold orientations, we normalized them by subtracting the scores from the pertinent condition without a handhold. For example, a person’s response direction in condition *no-vis* with *grip-body* was normalized by subtracting that person’s response direction in condition *no-vis* without a handhold. In what follows, *grip-body* and *grip-tilt* will refer to these normalized data.

Data registered without tactile information were submitted to an analysis of variance (ANOVA) with repeated measures on the factors Session (E, P) and Condition (*no-vis, vis-body, vis-tilt*). We also used those data to calculate the relative weight of visual cues, separately for each participant and session, using

1 $${{w}}_{\mathrm{v}} = \frac{{\left[ {{\mathrm{response}}\,{\mathrm{direction}}\,{\mathrm{in}}\,vis - body} \right]-[{\mathrm{response}}\,{\mathrm{direction}}\,{\mathrm{in}}\,vis - tilt]}}{{[{\mathrm{visual}}\,{\mathrm{cue}}\,{\mathrm{orientation}}\,{\mathrm{in}}\,vis - body]-[{\mathrm{visual}}\,{\mathrm{cue}}\,{\mathrm{orientation}}\,{\mathrm{in}}\,vis - tilt]}}.$$

The numerical value of the denominator in Eq. (1) was 45° in our study. Since the relative weight of gravicentric cues was zero in the present study, we calculated the relative weight of egocentric cues as *w*_e_ = 1 − *w*_v_. For purposes of comparison, we also calculated *w*_v_ and *w*_e_ for participants who assumed an upright rather than prone body posture on ground, using data from an earlier study.^30^ We will refer to those data as session E′. Thirty-five persons participated in that earlier study, and none of them was included again in the present study. We used again Eq. (1) to calculate *w*_v_. However, the relative weight of gravicentric cues *w*_g_ was nonzero in E′, and we therefore calculated *w*_e_ = 1 − *w*_v_ − *w*_g_, where

2 $${{w}}_{\mathrm{g}} = \frac{{\left[ {{\mathrm{response}}\,{\mathrm{direction}}\,{\mathrm{in}}\,vis - body\,{\mathrm{of}}\,{\mathrm{session}}\,{\mathrm{E}}^\prime } \right]-[{\mathrm{response}}\,{\mathrm{direction}}\,{\mathrm{in}}\,vis - body\,{\mathrm{of}}\,{\mathrm{session}}\,{\mathrm{T}}]}}{{[{\mathrm{body}}\,{\mathrm{orientation}}\,{\mathrm{in}}\,{\mathrm{session}}\,{\mathrm{E}}^\prime ]-[{\mathrm{body}}\,{\mathrm{orientation}}\,{\mathrm{in}}\,{\mathrm{session}}\,{\mathrm{T}}]}}.$$

In Eq. (2), session T refers to data collected while the participant was tilted 45° left-ear down, i.e., the numerical value of the denominator was again 45°. For statistical analysis, weights in session E and P were compared by two-sided, paired-samples *t* tests. Since our earlier study involved a different group of participants, weights in E′ were compared to those in E and to those in P by separate *t* tests for independent samples. *T* tests were Bonferroni-corrected for multiple testing.

Data registered with a handhold were submitted to an ANOVA with repeated measures on Session (E, P), Condition (*no-vis, vis-tilt*) and with the grouping factor Handhold (*grip-body, grip-tilt*). Data registered in the same persons with and without a kidney belt were submitted to an ANOVA with repeated measures on Session (E, P), Condition (*no-vis, vis-tilt*) and Belt (yes, no).

Standard deviations of response directions registered without a handhold were calculated for each participant, and were submitted to an ANOVA with repeated measures on the factors Session (E, P) and Condition (*no-vis*, *vis-body*, *vis-tilt*). In addition, we used an ANOVA with the grouping factor Session (E, E′) and with repeated measures on the factor Condition (*no-vis*, *vis-body*, *vis-tilt*), as well as an ANOVA with the grouping factor Session (P, E′) and with repeated measures on the factor Condition (*no-vis*, *vis-body*, *vis-tilt*).

### Reporting summary

Further information on research design is available in the Nature Research Reporting Summary linked to this article.

## Supplementary information

Reporting Summary Checklist

## Data Availability

The minimal dataset that would be necessary to interpret, replicate and build upon the findings reported in the present article is available to any reader directly upon reasonable request. Address the request to the first or second author.
